# Detection and Management of Geographic Atrophy Secondary to Age-Related Macular Degeneration Using Noninvasive Retinal Images and Artificial Intelligence: Systematic Review

**DOI:** 10.2196/81328

**Published:** 2025-11-21

**Authors:** Nannan Shi, Jiaxian Li, Mengqiu Shang, Weidao Zhang, Kai Xu, Yamin Li, Lina Liang

**Affiliations:** 1 Department of Eye Function Laboratory, Eye Hospital China Academy of Chinese Medical Sciences, No.33 Lugu Road, Shijingshan District, Beijing, 100040, China, +86 010-68683451; 2 Department of Ophthalmology, The First Affiliated Hospital of Yunnan University of Chinese Medicine, Yunnan Provincial Hospital of Traditional Chinese Medicine, Kunming, China

**Keywords:** geographic atrophy, dry age-related macular degeneration, artificial intelligence, noninvasive retinal images, systematic review

## Abstract

**Background:**

Geographic atrophy (GA), the endpoint of dry age-related macular degeneration (AMD), is irreversible. The recent approval by the Food and Drug Administration of a complement component 3 inhibitor marks a significant breakthrough, highlighting the critical importance of early detection and management of GA. Consequently, there is an urgent and unmet need for efficient, accurate, and accessible methods to identify and monitor GA. Artificial intelligence (AI), particularly deep learning (DL), applied to noninvasive retinal imaging, offers a promising solution for automating and enhancing GA management.

**Objective:**

This systematic review aimed to assess the performance of AI using noninvasive imaging modalities and compare it with clinical expert assessment as the ground truth.

**Methods:**

Two consecutive searches were conducted on PubMed, Embase, Web of Science, Scopus, Cochrane Library, and CINAHL. The last search was performed on October 5, 2025. Studies using AI for GA secondary to dry AMD via noninvasive retinal imaging were included. Two authors worked in pairs to extract the study characteristics independently. A third author adjudicated disagreements. Quality Assessment of Diagnostic Accuracy Studies-AI and Prediction Model Risk of Bias Assessment Tool (PROBAST) were applied to evaluate the risk of bias and application.

**Results:**

Of the 803 records initially identified, 176 were found through an updated search. Subsequently, 200 papers were assessed in full text, of which 41 were included in the final analysis, 10 for GA detection, 20 for GA assessment and progression, and 11 for GA lesion prediction. The reviewed studies collectively involved at least 24,592 participants (detection: n=7132, assessment and progression: n=14,064, and prediction: n=6706), with a wide age range of 50 to 94 years. The studies spanned a diverse array of countries, including the United States, the United Kingdom, China, Austria, Australia, France, Israel, Italy, Switzerland, and Germany, as well as a multicenter study encompassing 7 European nations. The studies used a variety of imaging modalities to assess GA, including color fundus photography, fundus autofluorescence, near-infrared reflectance, spectral domain–optical coherence tomography (OCT), swept-source (SS)-OCT, and 3D-OCT. DL algorithms (eg, U-Net, ResNet50, EfficientNetB4, Xception, Inception v3, and PSC-UNet) consistently showed remarkable performance in GA detection and management tasks, with several studies achieving performance comparable to clinical experts.

**Conclusions:**

AI, particularly DL-based algorithms, holds considerable promise for the detection and management of GA secondary to dry AMD with performance comparable to ophthalmologists. This review innovatively consolidates evidence across GA management—from initial detection to progression prediction—using diverse noninvasive imaging. It has strong potential to augment clinical decision-making. However, to realize this potential in real-world settings, future research is needed to robustly enhance reporting specifications, ensure data diversity across populations and devices, and implement rigorous external validation in prospective, multicenter studies.

## Introduction

Age-related macular degeneration (AMD) is a progressive retinal disorder affecting millions of people worldwide [1]. In its advanced stages, characterized by neovascularization and geographic atrophy (GA), it can lead to significant vision loss, although symptoms may be subtle during the early and intermediate phases [2]. The Classification of Atrophy Meetings group has defined atrophy lesion development as incomplete retinal pigment epithelium (RPE) and outer retinal atrophy and complete RPE and outer retinal atrophy (cRORA) based on imaging methods [3]. GA, also known as cRORA, is the endpoint of dry AMD and is characterized by the loss of photoreceptors, RPE, and choriocapillaris [4,5]. With the advent of 2 approved therapies for GA secondary to AMD in 2023, namely pegcetacoplan (Syfovre) [6] and avacincaptad pegol [7], the treatment of GA represents a significant breakthrough. However, the effectiveness of these therapies relies heavily on early detection and the ability to monitor treatment response—a significant unmet need in current clinical practice. The recent approval of complement inhibitors underscores the necessity for precise, reproducible, and practical tools to not only identify GA at its earliest stages but also to objectively track morphological changes over time, thereby evaluating therapeutic efficacy [8,9]. Artificial intelligence (AI) is uniquely positioned to address this gap by enabling precise, reproducible, and automated quantification of GA progression and treatment response using noninvasive imaging modalities [10]. Unlike conventional methods that rely on subjective and time-consuming manual assessments, AI algorithms can detect subtle subclinical changes in retinal structures—such as photoreceptor integrity loss, RPE atrophy, and hyperreflective foci—long before they become clinically apparent. Thus, AI-based retinal imaging offers a critical foundation for early detection and timely intervention in GA.

Various imaging techniques, both invasive and noninvasive, can directly visualize GA lesions. Invasive methods, such as fluorescence angiography, often result in a poor patient experience and entail high costs due to pupil dilation and sodium fluorescein injection. While it remains the gold standard for assessing neovascular AMD and offers significant diagnostic insights for retinal vascular diseases, in most cases, noninvasive fundus images are used for GA diagnosis and management [2]. Color fundus photography (CFP), fundus autofluorescence (FAF), and near-infrared reflectance (NIR) are based on 2D images, which can generally produce results to quantify the atrophic area but fail to identify the retinal structure axially [11]. Compared with fundus imaging, optical coherence tomography (OCT) provides high-resolution, noninvasive 3D images of retinal structures for macular assessment. In addition, conventional B-scan (axial direction) OCT images can be integrated with en-face scans, facilitating the identification of atrophy borders similar to FAF [10,12]. Nonetheless, manual labeling is tedious, time-consuming, and impractical in a clinical setup [13]. There is an urgent and unmet need for early detection and management of GA using retinal image modalities. Recent advancements in AI, especially deep learning (DL), present a promising opportunity for enhancing GA detection, classification, segmentation, quantification, and prediction.

In the 1950s, AI referred to computer systems capable of performing complex tasks that historically only a human could do. So what is AI? How is it used in medicine today? And what may it do in the future? AI refers to the theory and development of computer systems capable of performing tasks that historically required human intelligence, such as recognizing speech, making decisions, and identifying patterns. AI is an umbrella term that encompasses a wide variety of technologies, including machine learning (ML) and DL [14]. ML is a subfield of AI that uses algorithms trained on datasets to create self-learning models capable of predicting outcomes and classifying information without human intervention [15]. ML refers to the general use of algorithms and data to create autonomous or semiautonomous machines. DL, meanwhile, is a subset of ML that layers algorithms into “neural networks” with 3 or more layers. Thus, it somewhat resembles the human brain, enabling machines to perform increasingly complex tasks [16]. DL algorithms generally have high and clinically acceptable diagnostic accuracy across different areas (ophthalmology, respiratory, breast cancer, etc) in radiology [17]. Within ophthalmology, DL algorithms showed reliable performance for detecting multiple findings in macular-centered retinal fundus images [18]. Therefore, automatic GA segmentation plays a vital role in the diagnosis and management of advanced AMD and its application in the clinical setting.

Given the rapid evolution of AI applications in ophthalmology and the growing clinical importance of GA, this study aimed to systematically review the current evidence on AI-based approaches for the detection and management of GA secondary to dry AMD using noninvasive imaging modalities. We aimed to evaluate diagnostic accuracy relative to reference standards and examine methodological challenges to inform the design of future research and clinical implementation.

## Methods

### Protocol and Registration

Before starting this systematic review and meta-analysis, we registered a protocol on the PROSPERO website. This review adhered to the PRISMA (Preferred Reporting Items for Systematic Reviews and Meta-Analyses) and PRISMA-DTA (PRISMA of Diagnostic Test Accuracy) checklists [19,20].

### Eligibility Criteria

We included studies using AI algorithms to detect, classify, identify, segment, quantify, or predict GA secondary to AMD from CFP, OCT, OCT angiography, FAF, or NIR. The data were from participants, with or without symptoms, who were diagnosed with GA (or cRORA) secondary to nonexudative AMD. Study designs were not restricted; multicenter or single-center, prospective or retrospective, post hoc analysis, clinical study, or model development studies were all accepted. Eyes with neovascular complications or macular atrophy from causes other than AMD, any previous anti-vascular endothelial growth factor treatment, any confounding retinopathy, or poor image quality were excluded.

### Electronic Search Strategy

Two consecutive searches were conducted on PubMed, Embase, Web of Science, Scopus, Cochrane Library, and CINAHL. Because this review required the extraction of baseline data and items, considering the completeness of the data, we did not conduct any in press or print source searches and excluded conference proceedings and similar materials. The initial search was completed from the date of entry to December 1, 2024; the updated search, from December 1, 2024, to October 5, 2025. We used a search strategy for patient (GA) and index tests (AI and retinal images) that had been used in previous Cochrane Review without any search peer review process [21]. There were no restrictions on the date of publication. The language was limited to English. In Multimedia Appendix 1, detailed search strategies for each database are provided. During this process, no filters were used. During the search process, we adhered to the PRISMA-S (Preferred Reporting Items for Systematic reviews and Meta-Analyses literature search extension) reporting guidelines [22].

### Selection Process

All relevant literature was imported into EndNote (version 20; Clarivate Analytics) software, and literature screening was conducted independently by 2 researchers (NS and JL) who specialize in ophthalmology. Duplicates were removed from the software, and the titles and abstracts of the literature were reviewed to identify those relevant to the topic. Finally, the full texts were downloaded and examined, leading to the selection of literature that met the inclusion criteria. In cases of inconsistencies in the final inclusion decisions made by the 2 researchers, a third professional (LL) was consulted to resolve the dispute.

### Data Collection Process

Using standardized data items, the data were extracted independently from the included studies by 2 researchers (NS and JL). A third review author (LL) confirmed or adjudicated any discrepancies through group discussion. We retrieved the following data items: (1) study characteristics (author, year, study design, region, and theme), (2) dataset characteristics (databases, source of databases, training/validation/testing ratio, patient number, number of images or volumes, scan number, mean age, clinical registration number, and model evaluation method), (3) image and algorithm characteristics (devices, metrics, image modality, image resolution, and AI algorithms), (4) performance metrics (outcomes, performance of models, ground truth, and performance of the ophthalmologists), and (5) main results. All the information was retrieved from the main text and the tables provided in Multimedia Appendix 2. Therefore, we did not seek additional data by contacting the authors or experts. In some studies, the authors reported multiple sets of performance data based on a subset of a single dataset. For example, they may have reported results such as sensitivity, specificity, accuracy, and so forth, conducted on the cross-validation set, the test set, or the development set. We referred to the relevant literature to select the optimal set of test performance results [21]. However, when the primary study provided performance results based on a single test, the development dataset was used to train the AI model, and an external validation set ultimately was used to determine the performance of the optimal model. We extracted the external validation set performance data [23].

### Risk of Bias and Application

We worked in pairs to assess the risk of bias and the applicability of the studies, which involved detection, classification, identification, segmentation, and quantification using the Quality Assessment of Diagnostic Accuracy Studies (QUADAS)-AI [24] and the modified QUADAS-2 tool [25], while predictive studies used the Prediction Model Risk of Bias Assessment Tool (PROBAST) [26].

In the current context, QUADAS-AI has not yet established a complete specification of items. Therefore, we referenced the examples provided by QUASAS-AI and the published literature to compile the revised QUADAS-AI items, which included 4 domains and 9 leading questions (Table S4 in Multimedia Appendix 2). The PROBAST tool comprises participants, predictors, outcomes, and analysis, containing 20 signaling questions across 4 domains (Table S5 in Multimedia Appendix 2). We also evaluated the applicability of the study based on the leading or signaling questions in the first 3 domains. A study with “yes” answers to all index questions was considered to have a low risk of bias. If the answer to any of the informational questions was “no,” there was a potential for bias, leading the authors to rate the risk of bias as high. “Indeterminate” grades were applied when detailed content was not provided in the literature, making it difficult for the evaluator to reach a judgment. They were used only when the reported data were insufficient. Throughout the process, disagreements between the 2 reviewers (NS and JL) were resolved by consulting the senior reviewer (LL).

### Data Synthesis

As very few studies reported the number of true positives, true negatives, false positives, and false negatives, we restricted the quantitative analysis to determine the diagnostic accuracy of AI as a triaging tool for GA secondary to nonexudative AMD. However, a meta-analysis was not performed due to significant methodological heterogeneity across studies, arising from diverse AI architectures, imaging modalities, outcome metrics, and validation protocols. Instead, a systematic review was performed to qualitatively summarize performance trends. This approach allowed for a comprehensive evaluation of the AI capabilities in the detection and management of GA via noninvasive images.

## Results

### Study Selection

**Figure 1.**
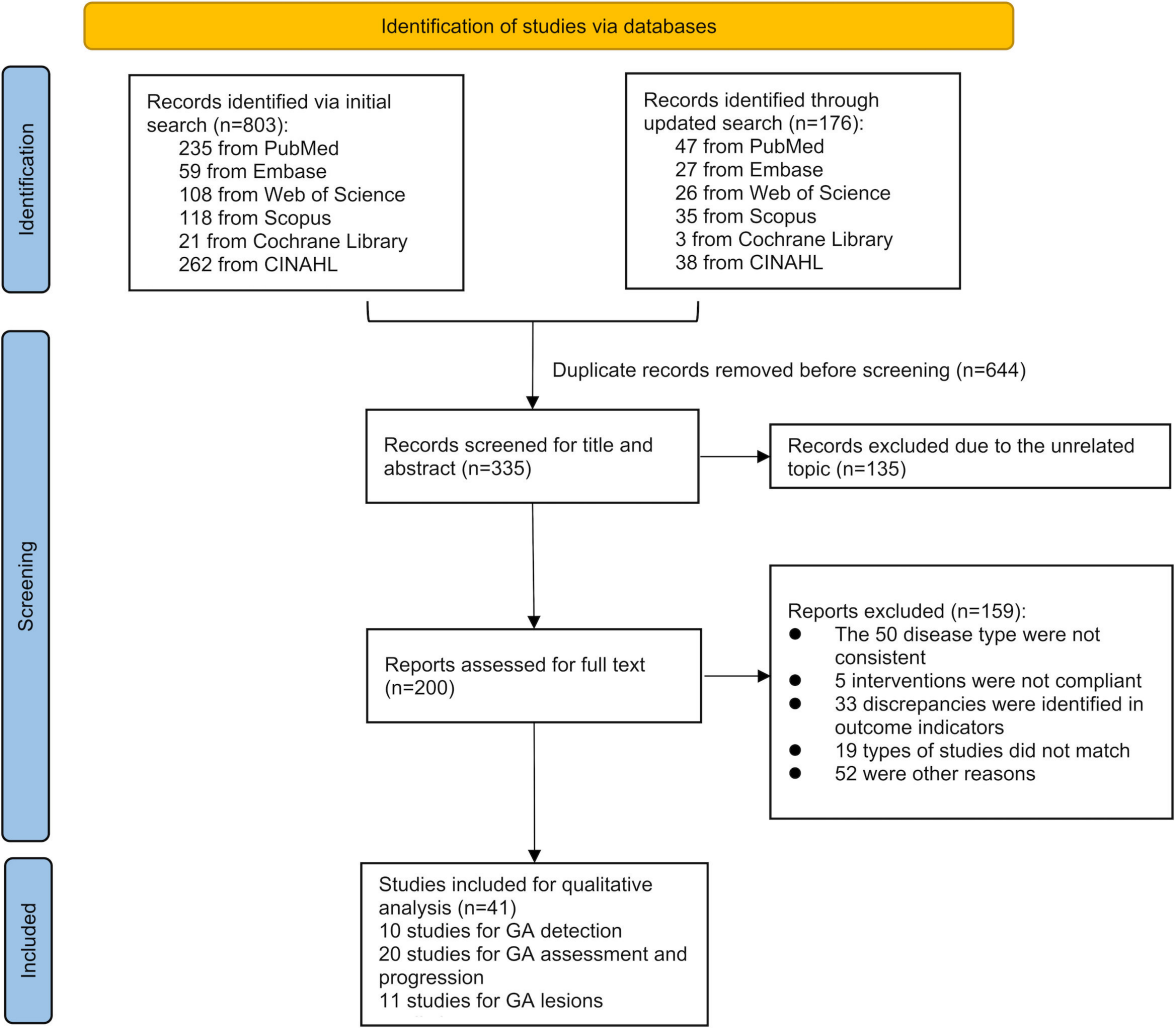
PRISMA (Preferred Reporting Items for Systematic Reviews and Meta-Analyses) flow diagram for literature selection. GA: geographic atrophy.

A total of 979 records related to the topic of this systematic review were searched across 6 different databases using a combination of subject terms and free-text terms. After removing duplicates, 335 records remained and were examined for titles and abstracts. Excluding studies not relevant to the research topic resulted in 200 reports. The full texts were then downloaded and reviewed in detail based on the eligibility criteria for the studies. In the final qualitative analysis, 41 studies were included. Of these, 10 focusing on GA diagnosis, 20 on GA assessment and progression, and 11 on GA prediction. Figure 1 presents the detailed flow diagram of the literature selection.

### AI in Detecting the Presence of GA

Ten of the 41 included studies focused on AI-based detection of GA using noninvasive retinal images (Table S1 in Multimedia Appendix 2). As listed in Table 1, the studies were published from 2018 to 2025. Four of the studies [27-30] focused on model development, 3 [31-33] were retrospective studies, and 3 [34-36] were prospective studies (1 multicenter cohort study, 1 multicenter and low-interventional clinical study, and 1 clinical study). Geographically, half were from the United States, with others from Israel, Italy, Switzerland, Germany, and a multicenter European collaboration. The studies addressed several detection-related tasks: 5 focused solely on GA detection [27-29,34,35], 2 covered detection and classification [30,33], and others integrated detection with quantification or segmentation [31,32,36].

**Table 1.** Characteristics of studies evaluating artificial intelligence (AI) models for geographic atrophy (GA) detection using noninvasive retinal imaging.

Author	Study design	Region	Purpose of the study	Source of datasets	Number of patients	Number of images or scans	Model evaluation method	Image modality (image resolution)	AI^a^ algorithms	Outcomes	Performance of models
Fineberg et al [33]	Retrospective cohort study	Israel (Petah Tikva)	Detection and classification (GA)	Rabin Medical Center	113	659	10-fold cross-validation	NIR (640*640 pixels)	CNNs:^d^ ResNet50, EfficientNetB0, ViT_B_16, and YOLOv8 variants.	ACC, *P*, SEN, SPE, *F*_1_, IoU^i^, and DSC^g^	GA classification: EfficientNetB0: ACC^b^=0.9148; *P*^m^=0.9204; SEN^p^=0.9233; SPE^q^=1.0; *F*_1_=0.9147. ResNet50: ACC=0.8815; *P*=.8933; SEN=0.8917; SPE=0.9833; *F*_1_=0.8812. ViT_B_16: ACC=0.963; *P*=.9632; SEN=0.9667; SPE=1.0; *F*_1_=0.9629. GA detection: YOLOv8-Large: SEN=0.91; *P*=0.91; IoU=0.84; DSC^g^=0.88.
Kalra et al [31]	Retrospective clinical study	United States (Cleveland)	Detection, quantification, and segmentation (presence of GA and pixel-wise GA area measurement)	the Cole Eye Institute of the Cleveland Clinic	341	900	triple-fold cross-validation	SD-OCT^o^ (256*256 pixels)	CNN^d^: U-Net	*F*_1_, ACC, *P,* R^n^, SEN, and SPE	GA detection- ACC=0.91, SEN=0.86, SPE=0.94, *F*_1_=0.87. GA segmentation: ACC=0.96, SEN=0.95, SPE=0.93, *F*_1_=0.82.
Derradji et al [32]	Retrospective clinical study	Switzerland (Lausanne)	Detection and segmentation (RORA)	An existing image database of the Medical Retina Department at Jules-Gonin Eye Hospital	57	62	5-fold cross-validation	SD-OCT (NR^j^)	CNN: U-Net	SEN, DSC, *P*, and Kappa	Grader 1: DSC: mean 0.881 (SD 0.074); Precision: mean 0.928 (SD 0.054); SEN: mean 0.850 (SD 0.119); Kappa: mean 0.846 (SD 0.072). Grader 2: DSC: mean 0.844 (SD 0.076); Precision: mean 0.799 (SD 0.133); SEN: mean 0.915 (SD 0.064); Kappa: mean 0.800 (SD 0.082).
de Vente et al [36]	Prospective multicenter and low-interventional clinical study (including cross-sectional and longitudinal study part)	20 sites in 7 European countries	Detection and quantification (cRORA^f^)	The MACUSTAR Study Cohort	168	143 (ZEISS); 167 (Spectrails)	NR	SD-OCT (512*650 pixels)	CNN: U-Net	SEN, SPE, PPV^l^, NPV^t^, and Kappa	ZEISS: SEN=0.6; SPE=0.964; PPV=0.375; NPV=0.985. Spectralis: SEN=0.625; SPE=0.974; PPV=0.714; NPV=0.961.
Sarao et al [35]	Prospective clinical study	Italy (Udine)	Detection (presence of GA)	the Istituto Europeo di Microchirurgia Oculare (IEMO) study	180	540	NR	CFP^e^ (NR)	CNN: Efficientnet_b2	SEN, SPE, ACC, *F*_1_, R, AUROC, and AUPRC^c^	SEN: 100% (95%CI 83.2%-100%); SPE=97.5% (95% CI 86.8%-99.9%); ACC=98.4%; *F*_1_=0.976; R=1; AUROC^r^=0.988 (95% CI 0.918-1); AUPRC^c^=0.952 (95%CI 0.719-0.994).
Keenan et al [34]	Multicenter and prospective cohort study	United States (Maryland)	Detection (presence of GA)	Age-Related Eye Disease Study (AREDS) dataset	4582	59,812	5-fold cross-validation	CFP (512 pixels)	CNN: inception v3	ACC, SEN, SPE, *P*, AUC, and Kappa	ACC=0.965 (95% CI 0.959-0.971); Kappa=0.611 (95% CI 0.533-0.689); SEN=0.692 (95% CI 0.560-0.825); SPE=0.978 (95% CI 0.970-0.985); Precision=0.584 (95% CI 0.491-0.676).
Yao et al [29]	Model development and evaluation	United States (California)	Detection (presence of nGA)	the Early Stages of AMD^s^ (LEAD) study	140	1884	5-fold cross-validation	SD-OCT (512*496 pixels)	CNN: ResNet18	SEN, SPE, ACC, *P*, and *F*_1_	SEN=0.76 (95% CI 0.67-0.84); SPE=0.98 (95% CI 0.96-0.99); PRE=0.73 (95% CI 0.54-0.89); ACC=0.97 (95% CI 0.95-0.98); *F*_1_=0.74 (95% CI 0.61-0.84).
Chiang et al [27]	Model development	United States (California)	Detection (complete retinal pigment epithelial and outer retinal atrophy (cRORA) in eyes with AMD)	University of Pennsylvania, University of Miami, and Case Western Reserve University; (2) Doheny Image Reading Research Laboratory, Doheny-UCLA (University of California Los Angeles Eye Centers)	71 (training); 649 (testing #1); 60 (testing #2)	188 (training); 1117 (testing #1)	5-fold cross-validation	SD-OCT (256*256 pixels)	CNN: ResNet18	SEN, SPE, PPV, NPV, AUROC, and AUPRC	SEN=0.909 (95% CI 0.778-1.000); SPE=0.553 (95% CI 0.394-0.703); PPV=0.541 (95% CI 0.375-0.707); NPV=0.913 (95% CI 0.778-1.000); AUROC=0.84 (95% CI 0.75-0.94); AUPRC=0.82 (95% CI 0.70-0.93).
Elsawy et al [28]	Model development	United States (Maryland)	Detection (explain decision making and compare methods)	The Age-Related Eye Disease Study 2 (AREDS2) Ancillary SD-OCT^k^ study from Devers Eye Institute, Emory Eye Center, Duke Eye Center, and the National Eye Institute	311	1284 scans	10-fold cross-validation	SD-OCT (128*128 or 224* pixels)	3D CNN: deep-GA-Net	ACC, *P*, R, *F*_1_, Kappa, AUROC, and AUPRC	ACC=0.93 (95% CI 0.92-0.94); Precision=0.90 (95% CI 0.88-0.91); Recall=0.90 (95% CI 0.89-0.92); *F*_1_ score=0.90 (95% CI 0.89-0.91); Kappa=0.80 (95% CI 0.77-0.83); AUROC=0.94 (95% CI 0.93-0.95); AUPRC=0.91 (95% CI 0.90-0.93).
Treder et al [30]	Model development	Germany (Muenster)	Detection and classification (GA)	Public database: ImageNet	400 (training); 60 (test set)	400 (training); 60 (test set)	NR	FAF^h^ (NR)	Deep CNN: self-learning algorithm	SEN, SPE, and ACC	Probability score: mean 0.981 (SD 0.048); SEN=100%; SPE=100%; ACC=100%.

a AI: artificial intelligence.

b ACC: accuracy.

c AUPRC: area under the precision-recall curve.

d CNN: convolutional neural network.

e CFP: color fundus photography.

f cRORA: complete retinal pigment epithelium and outer retinal atrophy.

g DSC: dice similarity coefficient.

h FAF: fundus autofluorescence.

i IoU: intersection over union.

j NR: not reported.

k OCT: optical coherence tomography.

l PPV: positive predictive value.

m P: precision.

n R: recall.

o SD-OCT: spectral domain OCT.

p SEN: sensitivity.

q SPE: specificity.

r AUROC: area under the receiver operating characteristic curve.

s AMD: age-related macular degeneration.

t NPV: negative predictive value.

Dataset configurations varied: 6 studies used training, validation, and test sets [30-33,35,36]; 3 used only training and test sets [27,28,34]; and 1 included a tuning set [29]. Collectively, these studies involved at least 7132 participants, with ages ranging from 50 to 85 years. Three studies were registered with ClinicalTrials.gov (NCT00734487, NCT01790802, and NCT03349801) [28,29,36]. Cross-validation methods included 5-fold (40% of studies) [27,29,32,34], 10-fold (20%) [28,33], and triple-fold (10%) [31]; 30% did not report validation details.

Spectral-domain (SD)–OCT was the most frequently used imaging modality (6/10 of studies) [27-29,31,32,36], followed by CFP (2/10) [34,35], and FAF or NIR (2/10 each) [30,33]. Most studies applied image preprocessing techniques—such as size standardization, orientation adjustment, intensity normalization, and noise reduction—to improve model performance. DL-based algorithms for GA detection have been developed for multiple image modalities. For example, Derradji et al [32] trained a convolutional neural networks (CNNs), U-Net, to predict atrophic signs in the retina, based on the EfficientNet-b3 architecture. Kalra et al [31] and de Vente et al [36] also trained a DL model based on U-Net. Yao et al [29] applied 3D OCT scans with ResNet18 pretrained on the ImageNet dataset, and Chiang et al [27] developed CNN (ResNet18) to improve computational efficiency. Elsawy et al [28] proposed Deep-GA-Net, a 3D backbone CNN with a 3D loss-based attention layer, and evaluated the effectiveness of using attention layers. Sarao et al [35] used a deep CNN, the EfficientNet_b2 model, which was pretrained on the ImageNet dataset and is well-known for its high efficiency and small size. Keenan et al [34] established their model using Inception v3, while Treder et al [30] performed a deep CNN, a self-learning algorithm, processing input data with FAF images.

A total of 14 performance sets were extracted from the 10 studies. Key metrics included sensitivity, specificity, accuracy, positive predictive value, negative predictive value, intersection over union, area under the receiver operating characteristic curve, area under the precision-recall curve, *F*_1_-score, precision, recall, Kappa, and dice similarity coefficient. Six OCT-based studies showed that DL models could detect GA with high accuracy, comparable to human graders [27-29,31,32,36]. Two studies using CFP also reported strong performance [34,35], while FAF- and NIR-based approaches demonstrated excellent repeatability and reliability [30,33].

We conducted a thorough evaluation of the 10 diagnostic studies’ methodological quality for the “participant selection,” “index test,” “reference standard,” and “flow and timing” domains at the study level (Table 2). None of the studies had an overall low or unclear risk of bias; instead, every study had a high risk of bias in at least 1 of the 4 domains. Regarding “patient selection,” only 4 studies [29,32,33,36] described the eligibility criteria; the rest did not report them. One study [30] used an open dataset (ImageNet) and did not include a test set. The small sample size of 4 studies [32,33,35,36] may have resulted in overfitting. In addition, 3 studies [29,30,32] did not report image formats and resolutions. Five studies [30,31,34-36] had a high risk of bias in participant selection because the included participants were not only GA secondary to dry AMD but also had other unrelated diseases. Regarding the “Index test,” only 1 algorithm was externally validated using a different dataset [27]; all other items were evaluated as low risk.

**Table 2.** Methodological quality and applicability assessment for studies on geographic atrophy (GA) detection using the revised Quality Assessment of Diagnostic Accuracy Studies–Artificial Intelligence (QUADAS-AI).

Study	Risk of bias				Concerns regarding applicability		
	Patient selection	Index test	Reference standard	Flow and timing	Patient selection	Index test	Reference standard
Chiang et al [27]	High risk	Low risk	Low risk	Low risk	Low risk	Low risk	Low risk
Elsawy et al [28]	High risk	High risk	Low risk	Low risk	Low risk	Low risk	Low risk
Kalra et al [31]	High risk	High risk	Low risk	Low risk	High risk	Low risk	Low risk
Keenan et al [34]	High risk	High risk	Low risk	Low risk	High risk	Low risk	Low risk
Sarao et al [35]	High risk	High risk	Low risk	Low risk	High risk	Low risk	Low risk
Yao et al [29]	High risk	High risk	Low risk	Low risk	Low risk	Low risk	Low risk
Treder et al [30]	High risk	High risk	Low risk	Low risk	High risk	Low risk	Low risk
Vente et al [36]	High risk	High risk	Low risk	Low risk	High risk	Low risk	Low risk
Derradji et al [32]	High risk	High risk	Low risk	Low risk	Low risk	Low risk	Low risk
Fineberg et al [33]	High risk	High risk	Low risk	Low risk	Low risk	Low risk	Low risk

### AI in GA Assessment and Progression

Twenty studies explored AI for GA assessment and progression using noninvasive imaging, published between 2019 and 2025 (Table S2 in Multimedia Appendix 2). As shown in Table 3, these studies covered 11 segmentation [5,11,37-45], 2 algorithm optimization [46,47], 3 AMD progression classification [48-50], and 3 combined tasks such as identification, segmentation, and quantification [51-53]. One study focused solely on GA quantification [54]. Retrospective analyses accounted for 9 studies [5,40,41,43,44,47,48,50,51], while 7 were model development [11,37-39,45,49,53], and the remainder were prospective [52,54], comparative [46], or cross-sectional [42]. Geographically, contributions came from China (6/20), the United States (7/20), the United Kingdom (2/20), Australia (2/20), France (1/20), Israel (1/20), and Austria (1/20).

**Table 3.** Characteristics of studies evaluating artificial intelligence (AI) models for geographic atrophy (GA) assessment and progression using noninvasive retinal imaging.

Author	Study design	Region	Purpose of the study	Source of datasets	Number of patients	Number of images or scans	Model evaluation method	Image modality (Image resolution)	AI algorithms	Outcomes	Performance of models
Pramil et al [5]	Retrospective review of images	United States (Boston)	Segmentation (GA lesions)	The “SWAGGER” cohort of the non-Exudative Age-Related Macular Degeneration (from New England Eye Center at Tufts Medical Center)	90	126	5-fold cross-validation	SS-OCT^a^ (500*500 pixels)	CNN^b^: U-Net	SEN^c^, SPE^d^, and DICE	SEN=0.95; SPE=0.91; DSC (vs G1): mean 0.92 (SD 0.11); DSC^e^ (vs G2): mean 0.91 (SD 0.12).
Siraz et al [50]	Retrospective comparative study	United States (North Carolina)	Classification (central and noncentral GA)	Atrium Health Wake Forest Baptist	104	355	NR^f^	SD-OCT^g^ (224*224 pixels)	CNNs: ResNet50, MobileNetV2, and ViT-B/16	AUROC^h^, *F*_1_, and ACC^i^	(CGA^ah^ vs NCGA^k^) ResNet50: AUROC: mean 0.545 (SD 0.004), *F*_1_: mean 0.431 (SD 0.00); ACC: mean 0.756 (SD 0.00). MobileNetV2: AUROC: mean 0.521 (SD 0.016), *F*1: mean 0.432 (SD 0.002); ACC: mean 0.756 (SD 0.00). ViT-B/16: AUROC: mean 0.718 (SD 0.002), *F*_1_: mean 0.602 (SD 0.004); ACC: mean 0.780 (SD 0.005).
Arslan et al [43]	Retrospective cohort clinical study	Australia (Victoria)	Segmentation (GA lesion area)	The Center for Eye Research Australia or a private ophthalmology practice diagnosed with GA	51	702	5-fold cross-validation	FAF^l^ (768*768 or 1536*1536 pixels)	CNN: U-Net	DSC, DSC loss, SEN, SPE, MAE^m^, ACC, R, and P	DSC: mean 0.9780 (SD 0.0124); DSC loss: mean 0.0220 (SD 0.0041); SEN: mean 0.9903 (SD 0.0041); SPE: mean 0.7498 (SD 0.0955); MAE: mean 0.0376 (SD 0.0184); ACC: mean 0.9774 (SD 0.0090); P: mean 9837 (SD 0.0116).
Hu et al [48]	Retrospective clinical study	China (Shenyang)	Classification (dry AMD^p^ progression phases)	Shenyang Aier Eye Hospital	338	3401	5-fold cross-validation	SD-OCT (NR)	CNNs: EfficientNetV2, DenseNet169, Xception, and ResNet50NF	ACC, SEN, SPE, *F*_1_, Macro-f1, and Kappa	ACC=97.31%; SEN=89.25%; SPE=98.80%; *F*_1_=91.21%; Macro-f1=92.08%; Kappa=95.45%.
Spaide et al [41]	Retrospective analysis and model comparison	United States (Washington)	Segmentation (GA lesion area)	The SWAGGER cohort from the New England Eye Center at Tufts Medical Center	87	126 scans	5-fold cross-validation	SS-OCT (NR)	CNN: U-Net	DSC	UNet-1: 0.82 (95% CI 0.78-0.86). UNet-Avg: 0.88 (95% CI 0.85-0.91). UNet-Drop: 0.90 (95% CI 0.87-0.93).
Vogl et al [47]	Retrospective analysis	Austria (Vienna)	Identification (GA progression after pegcetacoplan treatment)	The FILLY trial	156	NR	NR	SD-OCT (512*512 pixels)	CNN: U-Net	LPR^q^	Compared with sham treatment, monthly: −28% (−42.8 to −9.4). Every other month: −23.9% (−40.2 to −3.0).
Szeskin et al [51]	Retrospective analysis	Israel (Jerusalem)	Identification, quantification (GA lesion)	Datasets D1 and D2 from the Hadassah University Medical Center	D1: 18; D2: 16	NR	4-fold cross-validation	SD-OCT (496*1024 pixels and 496*1536 pixels)	CNN: the custom column classification CNN	AUROC, *P*, R^n^, and *F*_1_	AUROC=0.970; (Segment) P: mean 0.84 (SD 0.11); R: mean 0.94 (SD 0.03); (Lesion) P: mean 0.72 (SD 0.03); R: mean 0.91 (SD 0.18).
Spaide et al [40]	Retrospective analysis	United States (California)	Segmentation (GA lesion area)	Proxima A and B	Proxima A: 154; Proxima B: 183	Proxima A: 497; Proxima B: 940	NR	FAF, NIR^r^ (768 *768 pixels)	Multimodal DL^s^: U-Net; YNet	DSC and r^2t^	(G1-Ynet)DSC: mean 0.92 (SD 0.09). (G1-Unet)DSC: mean 0.90 (SD 0.09). (G2-Ynet)DSC: mean 0.91 (SD 0.09). (G2-Unet)DSC: mean 0.90 (SD 0.09). (Ynet) r^2^: 0.981. (Unet) r^2^: 0.959.
AI-khersan et al [44]	Retrospective analysis	United States (Texas)	Segmentation (GA)	The Retina Consultants of Texas and Retina Vitreous Associates	33; 326	367; 348	5-fold cross-validation	SD-OCT (512*496pixels; 200*1024pixels)	CNN: 3D-to-2D U-Net	DSC and r2	For Spectralis data, DSC=0.826; *r*^2^=0.906. For Cirrus data, DSC=0.824; *r*^2^=0.883.
Chu et al [52]	Prospective study	United States (Washington)	Identification, segmentation, and quantification (GA)	The University of Miami	70; 20; 25	NR	NR	SS-OCT (512*512 pixels)	CNN: U-Net	DSC, SEN, and SPE	DSC: mean 0.940 (SD 0.032). SEN=100%; SPE: 100%.
Merle et al [54]	Prospective observational study	Australia (Victoria)	Quantification (GA)	The Center for Eye Research Australia	50	NR	NR	SD-OCT; FAF (NR)	CNN: U-Net	Spearman correlation coefficient and SEN	(OCT^u^-automatically) Spearman correlation coefficient=0.85 (95% CI 0.71-0.91); SEN=0.59.
Yang et al [49]	Model development	China (Shenyang)	Classification (stage of dry AMD progression)	Shenyang Aier Excellence Eye Hospital	1310	16,384	3-fold cross-validation	SD-OCT (NR)	CNNs: ResNet50, EfficientNetB4, MobileNetV3, Xception	ACC, SEN, SPE, and *F*_1_	ACC(GA): ResNet50=92.35%; EfficientNetB4=93.85%; MobileNetV3=89.64%; Xception=91.16%. ACC (nascent GA): ResNet50=91.56%; EfficientNetB4=89.66%; MobileNetV3=89.43%; Xception=85.22%.
Ji et al [37]	Model development	China (Nanjing)	Segmentation (GA lesion area)	Dataset1 and dataset2	8; 54	NR	NR	SD-OCT (224*224 pixels)	Weakly supervised multitask learning: Mirrored X-Net	DSC, IoU^v^, AAD^w^, and CC^x^	DSC: mean 0.862 (SD 0.080); IoU: mean 0.765 (SD 0.119); AAD: mean 0.090 (SD 0.090); CC: 0.992.
Ma et al [38]	Model development	China (Jinan)	Segmentation (GA lesion area)	Dataset1 and dataset2	62	NR	5-fold cross-validation	SD-OCT (224*224 pixels)	Weakly supervised model: VGG16	DSC, OR^y^, AAD, CC, and AUROC	DSC: mean 0.847 (SD 0.087); OR: mean 0.744 (SD 0.126); AAD: mean 0.150 (SD 0.149); CC: 0.969; AUROC: 0.933.
Royer et al [39]	Model development	France (Issy-Les-Moulineaux)	Segmentation (GA lesion area)	the Clinical Imaging Center of the Quinze-Vingts Hospital	18	328	8 different random combinations of 12 series to train the model and 6 for the tests	NIR (256*256 pixels)	Unsupervised neural networks: W-net	*F*1, *P*, and R	*F*_1_: mean 0.87 (SD 0.07); *P*: mean 0.90 (SD 0.07); R: mean 0.85 (SD 0.11).
Xu et al [11]	Model development	China (Jinan)	Segmentation (GA lesion area)	dataset1 and dataset2	8 (test I); 56 (test II)	55 (dataset1); 56 (dataset2)	NR	SD-OCT (1024*512*128pixels; 1024*200*200pixels)	Self-learning algorithm	OR, AAD, and CC	OR: mean 84.48% (SD 11.98%); AAD: mean 11.09% (SD 13.61%); CC: 0.9948.
Zhang et al [53]	Model development	United Kingdom (London)	Segmentation and quantification (GA)	The FILLY study	200	984	NR	SD-OCT (NR)	CNN: U-Net	DSC, ICC^z^, ACC, SEN, SPE, and *F*_1_	Approach 1: ACC=0.91 (95% CI 0.89-0.93); *F*_1_=0.94 (95% CI 0.92-0.96); SEN=0.99 (95% CI 0.97-1.00); SPE=0.54 (95% CI 0.47-0.61); DSC: mean 0.92 (SD 0.14); ICC=0.94. Approach 2: ACC=0.94 (95% CI 0.92-0.96); *F*_1_=0.96 (95% CI 0.94-0.98); SEN=0.98 (95% CI 0.96-1.00); SPE=0.76 (95% CI 0.70-0.82); DSC: mean 0.89 (SD 0.18); ICC: 0.91.
Liu et al [45]	Model development	China (Wuhan)	Segmentation (GA)	Wuhan Aier Eye Hospital; the public dataset OCTA500	300	2923	5-fold cross-validation	SD-OCT (512*512 pixels)	Self-learning algorithm (dual-branch image projection network)	Jaccard index, DSC, ACC, *P*^o^, and R	DSC: mean 7.03 (SD 2.73); Jaccard index: mean 80.96 (SD 4.29); ACC: mean 91.84 (SD 2.13); P: mean 87.12 (SD 2.34); R: mean 86.56 (SD 2.92).
Williamson et al [42]	Cross-sectional study	United Kingdom (London)	Segmentation (GA lesion area)	INSIGHT Health Data Research Hub at Moorfields Eye Hospital	9875 (OCT); 81 (FAF)	NR	NR	3D-OCT; FAF (512*512 pixels)	Self-learning algorithm	PPV^aa^	0.86 (95% CI 0.79-0.92).
Safai et al [46]	Comparative analysis	United States (Wisconsin)	Identification (the best AI framework for segmentation of GA)	AREDS2^ab^ study; the GlaxoSmithKline (GSK) study	271(AREDS2); 100(GSK)	601 (AREDS2); 156 (GSK)	5-fold cross-validation	FAF (512*512 pixels)	CNNs: UNet, FPN^ac^, PSPNet, EfficientNet, ResNet, VGG^ad^, mViT^ae^	CC and DSC	FPN_EfficientNet: CC=0.98, DSC=0.931. FPN_CCesNet: CC=0.98, DSC=0.902. FPN_VGG: CC=0.98, DSC=0.934. FPN_mViT: CC=0.99, DSC=0.939. UNet_EfficientNet: CC=0.98, DSC=0.924. UNet_CCesNet: CC=0.97, DSC=0.930. UNet_VGG: CC=0.97, DSC=0.896; UNet_mViT: CC=0.99, DSC=0.938. PSPNet_EfficientNet: CC=0.93, DSC=0.890. PSPNet_CCesNet: CC=0.87, DSC=0.877. PSPNet_VGG: CC=0.95, DSC=0.900. PSPNet_mViT: CC=0.98, DSC=0.889.

a SS-OCT: swept-source OCT.

b CNN: convolutional neural network.

c SEN: sensitivity.

d SPE: specificity.

e DSC: dice similarity coefficient.

f NR: not reported.

g SD-OCT: spectral domain OCT.

h AUROC: area under the receiver operating characteristic curve.

i ACC: accuracy.

j CGA: central geographic atrophy.

k NCGA: noncentral geographic atrophy.

l FAF: fundus autofluorescence.

m MAE: mean absolute error.

n R: recall.

o P: precision.

p AMD: age-related macular degeneration.

q LPR: local progression rate.

r NIR: near-infrared reflectance.

s DL: deep learning.

t *r*2: Pearson correlation coefficient.

u OCT: optical coherence tomography.

v IoU: intersection over union.

w AAD: absolute area difference.

x CC: correlation coefficient.

y OR: overlap ratio.

z ICC: intraclass coefficient.

aa PPV: positive predictive value.

ab AREDS2: Age-Related Eye Disease Study 2.

ac FPN: Feature Pyramid Network.

ad VGG: Visual Geometry Group.

ae mViT: Mix Vision Transformer.

Dataset configurations varied: 9 out of 20 studies used training, validation, and test sets [40,41,43-46,50-52]; 11 studies used training and test sets [5,11,37-39,49]; 2 studies used training and validation sets [42,48]; 1 study comprised training, tuning, and internal validation sets [53]; and 2 studies did not specify [47,54]. Across studies, at least 14,064 participants provided image data for analysis. Less than half of the studies (9/20, 45%) provided demographic information, with the average age of participants ranging from 55 to 94 years. Six studies were registered with ClinicalTrials.gov (NCT01342926, NCT02503332, NCT02479386, NCT02399072, and NCT04469140 [5,40,41,46,47,53]). To assess the generalization ability of the DL model, cross-validation methods included 5-fold (8/20 studies [5,38,41,43-46,48]), 4-fold (1/20 study [51]), 3-fold (1/20 study [49]), and other approaches (1/20 study [39]). Nine studies did not report validation specifics.

Multiple imaging modalities supported GA assessment: spectral domain optical coherence tomography (SD-OCT) was most common, followed by swept-source OCT (SS-OCT), 3D-OCT, FAF, and NIR. Preprocessing techniques were widely applied to standardize images and improve model performance. Algorithm architectures varied, with U-Net being the most frequently used. Other approaches included custom CNNs, self-learning algorithms, weakly supervised models, and multimodal networks. For example, Hu et al [48] trained the DL models (ResNet-50, Xception, DenseNet169, and EfficientNetV2), evaluating them on a single fold of the validation dataset, with all *F*_1_-scores exceeding 90%. Yang [49] proposed an ensemble DL architecture that integrated 4 different CNNs, including ResNet50, EfficientNetB4, MobileNetV3, and Xception, to classify dry AMD progression stages. GA lesions on FAF were automatically segmented using multimodal DL networks (U-Net and Y-Net) fed with FAF and NIR images [40]. In contrast to the multimodal algorithms mentioned above (ie, the examples of DL models), Safai [46] investigated 3 distinct segmentation architectures along with 4 commonly used encoders, resulting in 12 different AI model combinations to determine the optimal AI framework for GA segmentation on FAF images.

From 20 studies, 42 performance sets were collected. Common metrics included correlation coefficient, mean absolute error, Spearman correlation coefficient, intraclass coefficient, overlap ratio, Pearson correlation coefficient (*r*^2^), Kappa, specificity (SPE), sensitivity (SEN), accuracy, positive predictive value (PPV), *F*_1_-score, P, R, intersection over union, and dice similarity coefficient (DSC). Regarding the segmentation, classification, identification, and quantification of GA in SD-OCT, 12 studies demonstrated performance comparable to that of clinical experts [11,37,38,44,45,47-51,53,54]. AI was also capable of efficiently detecting, segmenting, and measuring GA in SS-OCT, 3D-OCT, and FAF images, according to 4 studies [5,41,42,52]. AI for GA segmentation in FAF and NIR images, with clinical data showing good segmentation performance [39,40,43].

We performed a comprehensive assessment of the methodological quality of 16 GA assessment and progression studies encompassing 4 domains (Table 4). Only 8 studies detailed the eligibility criteria in the “patient selection” category, while the others had not been published. Three of the studies [47-49] lacked complete datasets, and 3 others [37,39,54] had small datasets or limited volumes of data. In addition, 3 studies [48,49,54] failed to provide information on image formats or resolutions. Two studies [48,49] were ranked as high risk regarding patient selection since the participants included other types of dry AMD (drusen, nascent GA). In terms of applicability, 18 studies were classified as low risk, while 2 were deemed high risk concerning patient selection. Concerning the “Index test,” only 3 algorithms underwent external validation with a different dataset [40,46,53]. All other items were evaluated as low risk.

**Table 4.** Methodological quality and applicability summary of geographic atrophy (GA) assessment and progression studies using revised Quality Assessment of Diagnostic Accuracy Studies–Artificial Intelligence (QUAUAS-AI).

Study	Risk of bias				Concerns regarding applicability		
	Patient selection	Index test	Reference standard	Flow and timing	Patient selection	Index test	Reference standard
M Hu [48]	High risk	High risk	Low risk	Low risk	High risk	Low risk	Low risk
JK Yang [49]	High risk	High risk	Low risk	Low risk	High risk	Low risk	Low risk
A Safai [46]	Low risk	Low risk	Low risk	Low risk	Low risk	Low risk	Low risk
WD Vogl [47]	High risk	High risk	Low risk	Low risk	Low risk	Low risk	Low risk
A Szeskin [51]	High risk	High risk	Low risk	Low risk	Low risk	Low risk	Low risk
ZD Chu [52]	High risk	High risk	Low risk	Low risk	Low risk	Low risk	Low risk
ZX Ji [37]	High risk	High risk	Low risk	Low risk	Low risk	Low risk	Low risk
X Ma [38]	High risk	High risk	Low risk	Low risk	Low risk	Low risk	Low risk
C Royer [39]	High risk	High risk	Low risk	Low risk	Low risk	Low risk	Low risk
T Spaide [40]	High risk	Low risk	Low risk	Low risk	Low risk	Low risk	Low risk
T Spaide [41]	High risk	High risk	Low risk	Low risk	Low risk	Low risk	Low risk
DJ Williamson [42]	Low risk	High risk	Low risk	Low risk	Low risk	Low risk	Low risk
RB Xu [11]	High risk	High risk	Low risk	Low risk	Low risk	Low risk	Low risk
J Arslan [43]	Low risk	High risk	Low risk	Low risk	Low risk	Low risk	Low risk
V Pramil [5]	Low risk	High risk	Low risk	Low risk	Low risk	Low risk	Low risk
GY Zhang [53]	High risk	Low risk	Low risk	Low risk	Low risk	Low risk	Low risk
DA Merle [54]	High risk	High risk	Low risk	Low risk	Low risk	Low risk	Low risk
H AI-khersan [44]	Low risk	High risk	Low risk	Low risk	Low risk	Low risk	Low risk
S Siraz [50]	Low risk	High risk	Low risk	Low risk	Low risk	Low risk	Low risk
XM Liu [45]	High risk	High risk	Low risk	Low risk	Low risk	Low risk	Low risk

### AI in Predicting GA Lesion Area and Progression

Eleven studies used AI for predicting GA lesion growth and progression using noninvasive imaging (Table S3 in Multimedia Appendix 2). These studies were published between 2021 and 2025, with some information provided in Table 5. The study designs consisted of 6 retrospective studies [55-60], 2 model development studies [61,62], 2 post hoc analyses [63,64], and 1 clinical evaluation of a DL algorithm [65]. Participants or images came from various regions: 6 studies were based in the United States [55,57-60,62], 3 in Australia [63-65], 1 in Switzerland [56], and another involving multiple centers in China and the United States [61]. Research aims focused on GA growth prediction [55,56,59-61,63,65], combined prediction and evaluation of lesion features [57], treatment response assessment [58], and integrated segmentation-prediction tasks [62,64].

**Table 5.** Characteristics of studies evaluating artificial intelligence (AI) models for geographic atrophy (GA) prediction using noninvasive retinal imaging.

Author	Study design	Region	Purpose of the study	Source of datasets	Number of patients	Number of images or scans or cubes	Model evaluation method	Image modality (resolution)	AI algorithms	Outcomes	Performance of models
Gigon et al [56]	Retrospective monocentric study	Switzerland (Lausanne)	Prediction (RORA^a^ progression)	Jules Gonin Eye Hospital	119	NR	NR^b^	SD-OCT^c^ (384*384 pixels)	CNN^d^: EfficientNet-b3	DSC^e^	0-6 months: 0.84 6-12 months: 0.84 >12 months: 0.89
Dow et al [55]	Retrospective cohort study	United States (Atlanta, Georgia, Portland, Oregon, North Carolina; Maryland, Raleigh, Morrisville, Cary); United Kingdom (Durham, South Durham)	Prediction (iAMD^f^ to GA within 1 year)	3 independent datasets from AREDS2^g^ and a tertiary referral center and associated satellites	316; 53; 48	1085; 53; 48	5-fold cross-validation	SD-OCT (512 *1000 pixels)	CNN: Inception v3	SEN^h^, SPE^i^, PPV^j^, NPV^k^, ACC^l^	SEN: 0.91 (95% CI 0.74-0.98); SPE: 0.80 (95% CI 0.63-0.91); PPV: 0.78 (95% CI 0.70-0.85); NPV: 0.92 (95% CI 0.90-0.95); ACC: 0.85 (95% CI 0.87-0.91)
Cluceru et al [57]	Retrospective clinical study; observation study	United States (California)	Prediction and evaluation (GA growth rate and GA features related to shape and size)	The lampalizumab phase 3 clinical trials and an accompanying observational study	1041; 255	NR	5-fold cross-validation	FAF^m^ (384 * 384 pixels)	CNN: VGG16	*r* ^2^ ^n^	Full FAF images: 0.44 (95% CI 0.36-0.49) Rim only: 0.37 (95% CI 0.35-0.4) Lesion only: 0.34 (95% CI 0.31-0.36) Background only: 0.3 (95% CI 0.27-0.33) Mask only: 0.27 (95% CI 0.24-0.29)
Anegondi et al [58]	Retrospective clinical study; observation study	United States (California)	Prediction and prognosis (GA lesion area and GA growth rate after lampalizumab treatment)	The lampalizumab phase 3 clinical trials and an accompanying observational study	1279; 443; 106; 169	NR	5-fold cross-validation	SD-OCT, FAF (512*512 pixels)	CNN: Inception v3	*r* ^2^	GA prediction: FAF-only: 0.98 (95% CI 0.97‐0.99) OCT-only: 0.91 (95% CI 0.87‐0.95), Multimodal: 0.94 (95% CI 0.92‐0.96). GA growth rate: FAF-only: 0.65 (95% CI 0.52‐0.75), OCT-only: 0.36 (95% CI 0.29‐0.43), Multimodal: 0.47 (95% CI 0.40‐0.54)
Salvi et al [59]	Retrospective analysis	United States (California)	Prediction (the 1 year region of growth of GA lesions)	The following lampalizumab clinical trials and prospective observational studies	597	NR	NR	FAF (768*768 pixels or 1536*1536 pixels)	CNN: U-Net	P^o^, R^p^, DSC, r2	Whole lesion: P: mean 0.70 (SD 0.12); R: mean 0.73 (SD 0.12); DSC: mean 0.70 (SD 0.09); r2: 0.79
Yoshida [60]	Retrospective analysis	United States (California)	Prediction (GA progression)	Three prospective clinical trials	1219; 442	NR	5-fold cross-validation	3D OCT (496*1024*49 voxels)	CNNs: (1) en-face intensity maps; (2) SLIVER-net; (3) a 3D CNN; and (4) en-face layer thickness and between-layer intensity maps from a segmentation model	*r* ^2^	GA lesion area: En-face intensity map: 0.91; SLIVER-net: 0.83; 3D DenseNet: 0.90; OCT EZ^q^ and RPE^r^ thickness map: 0.90; GA growth rate: En-face intensity map: 0.33; SLIVER-net: 0.33; 3D DenseNet: 0.35; OCT EZ and RPE thickness map: 0.35.
GS Reiter [63]	Post hoc analysis	Austria (Vienna)	Prediction (GA lesions progression)	the phase II randomized controlled trial FILLY	134	268 scans	5-fold cross-validation	FAF, NIR^s^, SD-OCT (NR)	CNN: PSC-UNet	ACC, Kappa, concordance index	ACC: 0.48; Kappa: 0.23; concordance index: 0.69
J Mai [64]	Post hoc analysis	Austria (Vienna)	Segmentation, quantification, and prediction (GA lesion and progression)	The phase 2 FILLY clinical trial and the Medical University of Vienna (MUV)	113; 100	226; 967	5-fold cross-validation	SD-OCT, FAF (768*768 and 1536*1536 pixels)	CNN: U-Net	DSC, Hausdorff distance, ICC^t^	MUV: DSC: mean 0.86 (SD 0.12); Hausdorff distance: mean 0.54 (SD 0.45); FILLY: DSC: mean 0.91 (SD 0.05); Hausdorff distance: mean 0.38 (SD 0.40)
YH Zhang [61]	Model development	China (Nanjing); United States (California)	Prediction (GA growth)	The Byers Eye Institute of Stanford University; the Jiangsu Provincial People’s Hospital	22; 3	86 cubes; 33 cubes	Leave-one-out cross-validation	SD-OCT (178*270 pixels)	Recurrent neural network: the bi-directional long-short term memory network; CNN: 3D-UNet	DSC, CC^u^	Scenario I: DSC: 0.86; CC: 0.83; Scenario II: DSC: 0.89; CC: 0.84; Scenario III: DSC: 0.89; CC: 0.86; Scenario IV: DSC: 0.92; CC: 0.88; Scenario V: DSC: 0.88; CC: 0.85; Scenario VI: DSC: 0.90; CC: 0.86
SX Wang [62]	Model development	United States (California)	Segmentation and prediction (GA lesion area and GA progression)	The University of California—Los Angeles	147	NR	8-fold cross-validation	SD-OCT, FAF (512*512 pixels)	CNN: U-Net	SEN, SPE, ACC, OR^v^	ACC: 0.95; SEN: 0.60; SPE: 0.96; OR: 0.65
J Mai [65]	Clinical evaluation of a DL-based algorithm	Austria (Vienna)	Prediction (GA lesions progression)	The Medical University of Vienna	100	967	5-fold cross-validation	SD-OCT, FAF (NR)	CNN: PSC-UNet	DSC, MAE^w^, and r2	0-1 year: DSC: mean 0.25 (SD 0.16); MAE: mean 0.13 (SD 0.11) 1-2 years: DSC: mean 0.38 (SD 0.20); MAE: mean 0.25 (SD 0.24); 2-3 years: DSC: mean 0.38 (SD 0.21); MAE: mean 0.35 (SD 0.34); >3 years: DSC: mean 0.37 (SD 0.23); MAE: mean 0.72 (SD 0.48)

a RORA: retinal pigment epithelial and outer retinal atrophy.

b NR: not reported.

c OCT: optical coherence tomography.

d CNN: convolutional neural network.

e DSC: dice similarity coefficient.

f AMD: age-related macular degeneration.

g AREDS2: Age-Related Eye Disease Study 2.

h SEN: sensitivity.

i SPE: specificity.

j PPV: positive predictive value.

k NPV: negative predictive value.

l ACC: accuracy.

m FAF: fundus autofluorescence.

n *r*2: Pearson correlation coefficient.

o P: precision.

p R: recall.

q EZ: ellipsoid zone.

r RPE: retinal pigment epithelium.

s NIR: near-infrared reflectance.

t ICC: intraclass coefficient.

u CC: correlation coefficient.

v OR: overlap ratio.

w MAE: mean absolute error.

Dataset structures varied: 3 out of 11 studies used training-validation-test splits [59,62,64]; 2 out of 11 studies used training-test sets [56,61]; 3 out of 11 studies used training-validation sets [55,63,65]; and the rest adopted development–holdout [57,60] or development-holdout-independent test configurations [58]. In total, 6706 participants were included across studies. Fewer than half of the studies (4/11, 36.4%) reported demographic information, with mean age ranges spanning from 74 to 83 years [55,58,64,65]. Six studies [57-60,63,64] were ethically approved and registered on ClinicalTrials.gov under the following identifiers: NCT02503332, NCT02247479, NCT02247531, NCT02479386, NCT01229215, and NCT02399072. The DL model’s generalizability was assessed using leave-one-out cross-validation in 1 study [61], 5-fold cross-validation in 7 studies [55,57,58,60,63-65], and 8-fold cross-validation in 1 study [62]. The remaining 2 studies [56,59] did not specify the cross-validation methodology.

Studies used 3D-OCT, SD-OCT, NIR, and FAF images, primarily sourced from Heidelberg, Zeiss, and Bioptigen devices. While most reported image metrics, 2 studies did not specify resolution details [63,65]. Commonly used DL architectures included Inception v3 [55,58], PSC-UNet [63,65], U-Net [59,62,64], EfficientNet-b3 [56], and VGG16 [57]. In addition, some studies introduced novel approaches, such as en-face intensity maps, SLIVER-net, 3D CNN, and a recurrent neural network, for improved GA progression forecasting.

According to various image modalities, datasets, and follow-up durations, we gathered 31 sets of performance data from 11 studies. The performance metrics included the Hausdorff distance, concordance index, overlap, SEN, SPE, accuracy, mean absolute error, Kappa, DSC, P, PPV, R, *r*^2^, and negative predictive value. The findings for a single image modality (3D-OCT, SD-OCT, or FAF) demonstrated the development of DL algorithms to predict GA growth rate and progression with excellent performance characteristics comparable to trained experts [55-57,59-61]. Multimodal approaches combining FAF, NIR, and SD-OCT further showed feasibility for individualized lesion growth prediction and localization [58,62-65].

In this systematic review, we used the PROBAST tool to rigorously evaluate prediction models across 4 domains, addressing 20 signaling questions for each paper reviewed. Within the “participants” domain, all studies used appropriate data sources; however, only 6 studies [57-60,64,65] clearly outlined their inclusion and exclusion criteria for participants, leaving the others unclear. In terms of “predictors,” these were defined and evaluated similarly for all participants, having no connection to outcome data and being available at baseline. All studies evaluated “yes” to the questions on outcome measurement methods, definitions, interference factors, and measurement time intervals. Concerning “analysis,” Dow [55] and Zhang [61] applied a small dataset with an insufficient number of participants. While Zhang performed internal validation, the lack of external validation notably limits the model’s generalizability, which was constructed with bi-directional long-short term memory networks and CNN frameworks. Two studies by Salvi [59] and Yoshida [60] lacked independent and external validation. Gigon [56] failed to explicitly mention missing data handling, complex problems, and model overfitting. Conversely, all other items were evaluated as low risk, and the applications of the studies were universally ranked as low risk (Table S1 in Multimedia Appendix 3).

## Discussion

### Principal Findings

This systematic review evaluated the performance of AI, particularly DL algorithms, in detecting and managing GA secondary to dry AMD using noninvasive imaging modalities. Our findings demonstrate that AI models exhibit strong capabilities in accurately detecting, segmenting, quantifying, and predicting GA progression from OCT, FAF, CFP, and NIR imaging, achieving diagnostic accuracy comparable to that of human experts. However, this review also identified several methodological challenges, such as limited sample sizes, inconsistent annotation standards, and a general lack of external validation, which may hinder the clinical generalizability and practical application of these models. Despite these limitations, AI-based tools show significant potential for future use by both specialists and nonspecialists in primary and specialty care settings.

### AI in Detecting GA With OCT, FAF, NIR, and CFP Images

Ten studies published between 2018 and 2025 were included, involving at least 7132 participants aged 50 to 85 years. Half of the studies were conducted in the United States, while others originated from European countries. SD-OCT was the most frequently used imaging modality (6/10 studies), followed by CFP (2/10 studies), NIR (1/10 studies), and FAF (1/10 studies). Image preprocessing techniques, such as standardization of size, orientation, and intensity, as well as noise reduction, were consistently applied to enhance model stability and training efficiency. However, 3 studies did not report critical image parameters, such as resolution, potentially limiting reproducibility. DL-based algorithms, including CNNs, were the primary methodologies used for GA detection. Cross-validation techniques, such as 5-fold and 10-fold methods, were used in half of the studies to assess model robustness, though 3 studies did not report validation strategies. AI, particularly DL algorithms, holds significant promise for the detection of GA using noninvasive imaging modalities. OCT, CFP, NIR, and FAF each demonstrated robust diagnostic potential, with performance metrics rivaling or exceeding human expertise.

### AI for GA Management With OCT, FAF, and NIR Images

A total of 20 studies (14,064 participants) were published between 2019 and 2025, focusing on themes such as GA segmentation, classification, quantification, and progression prediction. The research designs and geographic regions are diverse. The studies included retrospective analysis (9/20), model development (7/20), and prospective, comparative, or cross-sectional studies (4/20). Significant contributions came from China (6/20) and the United States (7/20), with additional studies from the United Kingdom (2/20), Australia (2/20), France (1/20), Israel (1/20), and Austria (1/20). The studies used a variety of imaging modalities to assess GA, including SD-OCT, FAF, NIR, SS-OCT, and 3D-OCT. DL algorithms demonstrated remarkable performance in GA management tasks. U-Net was the most commonly used architecture. Multimodal approaches combined FAF and NIR images with DL networks to improve segmentation accuracy. Performance metrics, such as DSC, Kappa, SEN, SPE, and accuracy, consistently showed strong diagnostic accuracy, with several studies achieving performance comparable to clinical experts.

Eleven studies with 6706 participants, published between 2021 and 2025, concentrated on the application of AI for predicting and segmenting GA lesions, as well as their growth and progression. The methodologies were diverse, including retrospective studies, model development studies, post hoc analyses, and clinical algorithm assessment. Participants or images were gathered from regions such as the United States, Australia, Switzerland, and various centers in China and the United States, ensuring broad geographic representation. Demographic information was reported in fewer than half of the studies, with a mean age ranging from 74 to 83 years. Imaging modalities, such as 3D-OCT, SD-OCT, NIR, and FAF, were obtained from devices including Bioptigen, Heidelberg Spectralis HRA+OCT, and Cirrus OCT. While the image preprocessing parameters were consistent across most studies, some did not specify image resolution. Multiview CNN architectures and advanced frameworks, such as the bi-directional long-short term memory networks, were used. DL algorithms exhibited excellent predictive capabilities, with multimodal approaches enabling individualized GA lesion growth prediction.

### Noninvasive Image Analysis Techniques for GA

GA, a late-stage form of dry AMD, is marked by the irreversible loss of photoreceptors, RPE, and choriocapillaris [4,5]. The application of noninvasive imaging modalities has revolutionized the detection and management of GA. A comparative summary of AI performance across these modalities is provided in Table S2 in Multimedia Appendix 3. CFP serves as a standard initial assessment tool, useful for screening and early detection. It identifies GA lesions as visible underlying choroidal vessels and well-defined regions of RPE hypopigmentation [66]. FAF imaging using a blue excitation wavelength (488 nm) visualizes metabolic changes at the level of photoreceptor or RPE complex and is practical in assessing GA lesion size and progression with hypo-autofluorescence [67]. In contrast to nonatrophic areas, GA lesions on NIR (787-820 nm, longer than FAF) typically appear brighter and less harmful to the eye [68]. In addition, NIR can help detect the boundaries of foveal lesions, where image contrast is lower on FAF [68]. Recently, the Classification of Atrophy Meeting group recommended that atrophy in both patients with and those without neovascular AMD be defined based on specific drusen characteristics and other anatomical features, and it is most easily characterized by OCT [69,70]. OCT stands out as the gold standard for GA detection and classification, providing high-resolution, cross-sectional, and en face images of the retina and choroid. SD-OCT is widely used in research and clinical trials, offering precise measurement of GA area and growth rates, while SS-OCT and 3D-OCT offer superior structural insights and potential for AI-driven automation [41,71,72]. Despite the higher cost and technical complexity of advanced OCT technologies, their detailed GA assessment capabilities make them indispensable tools in both clinical practice and research. Furthermore, OCT provides volumetric (3D) structural data, unlike the 2D en face projections of FAF, CFP, and NIR. It allows AI to learn not just the surface appearance of atrophy but also the cross-sectional structure alterations that define and precede GA [3]. As technology advances, the integration of AI and further developments in imaging techniques are expected to enhance the utility of these modalities, overcoming current limitations and expanding their applications in ophthalmology.

### Advantages and Challenges of AI Architectures in Clinical Workflow

AI addresses critical limitations of traditional GA monitoring, such as labor-intensive manual grading and intergrader variability [73]. Therefore, automated algorithms enable rapid, standardized analysis of large fundus image datasets, reducing clinician workload and enhancing reproducibility [74]. Furthermore, our review revealed a clear trend in the choice of model architectures tailored to specific clinical tasks. A critical analysis of these architectures is provided in Table S3 in Multimedia Appendix 3. Interestingly, with the advancement of AI algorithm architectures, numerous studies have emerged that use these technologies to identify atrophy caused by various retinal diseases and to evaluate treatment outcomes through image analysis. Miere et al [75] pretrained a DL-based classifier to automatically distinguish GA from atrophy secondary to inherited retinal diseases on FAF according to etiology, using 2 approaches (a trained and validated method and a 10-fold cross-validation method), achieving good accuracy and excellent area under the receiver operating characteristic (AUROC) values. In addition, a study examined the association between treatment and changes in photoreceptor lamina thickness in patients with GA secondary to AMD. The effect of pegcetacoplan on photoreceptors in OCT was supported by this post hoc analysis, which demonstrated that treatment with the drug was linked with reduced outer retinal thinning [76]. Similarly, DL-based OCT image analysis assessed the therapeutic effectiveness of complement component 3 inhibition in delaying GA progression, with findings indicating decreased photoreceptor thinning and loss [77]. Recent studies demonstrating the application of AI algorithms in imaging further validate their potential as reliable supplements to human expertise in the diagnosis and management of GA.

### Technical Challenges and Limitations

Despite the promising advancements in AI for GA detection and management, several technical challenges and limitations persist. A significant limitation of OCT-based AI models is their difficulty in distinguishing GA secondary to AMD from other forms of retinal atrophy; thus, the findings may not generalize to broader AMD cases or other retinal diseases, which limits their clinical applicability. In addition, images from different OCT devices show significant variability and imprecision, not offering good enough data acquisition [74]. Another major challenge is the variability in algorithm performance caused by differences in training data, image acquisition protocols, and disease definitions. These differences reduce reproducibility and limit practical deployment. For instance, the absence of standardized reporting in AI studies can result in discrepancies when interpreting results and hinder comparisons between different models. Moreover, despite the high-performance metrics (eg, SEN, SPE, DSC>0.85, and AUROC>0.95) reported by many studies, methodological limitations remain. All diagnostic studies included in this review were assessed as high risk in at least 1 domain (10/10), only 1 GA assessment study (1/20) was evaluated as low risk across all domains, and several prediction studies (7/11) were ranked as high or unclear risk in at least 1 domain, primarily due to small or nonrepresentative datasets and a lack of detailed reporting on image preprocessing and external validation. These methodological shortcomings may lead to an overestimation of AI model performance and reduced overall robustness, thereby decreasing the generalizability of the findings and limiting confidence in their real-world applicability. Future studies should prioritize the use of larger, more diverse datasets and implement rigorous validation frameworks to enhance performance metrics (including detection, segmentation, quantification, and prediction accuracy) and conduct prospective, multicenter validation studies to improve clinical applicability and generalizability. Furthermore, adherence to established reporting guidelines for AI studies (such as the Standards for Reporting Diagnostic Accuracy-AI and Checklist for Artificial Intelligence in Medical Imaging [78,79]) would improve comprehension and transparency, allow for more meaningful comparisons between systems, and facilitate meta-analyses.

### Real-World Implications and Research Contributions

Overall, despite some limitations, AI is constantly evolving and holds great potential for transformation in the health care sector [80]. AI has the potential to accelerate existing forms of medical analysis; however, its algorithms require further testing to be fully trusted. Clinically, AI-based automated tools show strong potential to facilitate early detection, precise quantification, progression, and prediction of GA, thereby reducing the burden on retinal specialists and improving diagnostic consistency. Furthermore, DL algorithms have demonstrated effectiveness in identifying retinal image features associated with cognitive decline, dementia, Parkinson disease, and cardiovascular risk factors [81]. These findings indicate that AI-based retinal images hold promise for transforming primary care and systemic disease management. Although most AI applications remain in the validation phase, the integration of AI with multimodal imaging, novel biomarkers, and emerging therapeutics holds promise for transforming clinical management paradigms in GA and advancing personalized medicine. Future efforts should focus on developing standardized datasets, improving algorithmic generalizability, and conducting real-world validation studies to fully integrate AI into routine ophthalmic practice.

### Conclusion

AI, especially DL-based algorithms, holds considerable promise for the detection and management of GA secondary to dry AMD, with performance comparable to trained experts. This systematic review synthesizes and critically appraises the current evidence, highlighting that AI’s capabilities extend across GA management—from initial detection and precise segmentation to the forecasting of lesion progression, which informs future research directions. Meanwhile, with the development of C5 inhibitors, AI-based noninvasive fundus image analysis is expected to detect, identify, and monitor GA at an early stage, thereby increasing the window of opportunity in the future. AI has strong potential to augment and streamline clinical workflows by offering automated, reproducible analysis that can assist clinicians in managing large volumes of imaging data; however, more studies are needed to further validate its effectiveness, repeatability, and accuracy.

## Supplementary material

10.2196/81328 Multimedia Appendix 1 Search strategies in all databases.

10.2196/81328 Multimedia Appendix 2 Summary of studies on geographic atrophy (GA) detection, GA assessment, GA prediction, quality assessment using Quality Assessment of Diagnostic Accuracy Studies–Artificial Intelligence (QUADAS-AI), and risk of bias evaluation using the Prediction Model Risk of Bias Assessment Tool (PROBAST).

10.2196/81328 Multimedia Appendix 3 Risk of bias and applicability assessment for geographic atrophy (GA) prediction studies, comparative performance of artificial intelligence (AI) models and features across imaging modalities for GA detection and management, and comparison of AI model architectures in GA detection and management.

10.2196/81328 Checklist 1 PRISMA 2020 checklist.

10.2196/81328 Checklist 2 PRISMA-S checklist.

## Abbreviations

AI: artificial intelligence
AMD: age-related macular degeneration
AUROC: area under the receiver operating characteristic curve
CFP: color fundus photography
CNN: convolutional neural network
cRORA: complete retinal pigment epithelium and outer retinal atrophy
DL: deep learning
DSC: dice similarity coefficient
FAF: fundus autofluorescence
GA: geographic atrophy
ML: machine learning
NIR: near-infrared reflectance
OCT: optical coherence tomography
PPV: positive predictive value
PRISMA: Preferred Reporting Items for Systematic Reviews and Meta-Analyses
PRISMA-DTA: Preferred Reporting Items for Systematic Reviews and Meta-Analysis of Diagnostic Test Accuracy
PRISMA-S: Preferred Reporting Items for Systematic Reviews and Meta-Analyses literature search extension
PROBAST: Prediction Model Risk of Bias Assessment Tool
QUADAS: Quality Assessment of Diagnostic Accuracy Studies
RPE: retinal pigment epithelium
SD-OCT: spectral domain optical coherence tomography
SEN: sensitivity
SPE: specificity
SS-OCT: swept-source OCT
