# Supplementary material for: Detection and Management of Geographic Atrophy Secondary to Age-Related Macular Degeneration Using Noninvasive Retinal Images and Artificial Intelligence: Systematic Review
Source: J Med Internet Res. 2025 Nov 21;27:e81328. doi: 10.2196/81328 (PMC12637997; doi:10.2196/81328)
Supplement: Multimedia Appendix 1 [file jmir-v27-e81328-s001.docx]

**1. PubMed search strategy**

((((((((((((("Artificial Intelligence"[Mesh]) OR (artificial intelligence)) OR (Computer Reasoning)) OR (AI)) OR (Machine Intelligence)) OR (Computational Intelligence)) OR (Computer Vision System)) OR (Knowledge Acquisition (Computer))) OR (Knowledge Representation (Computer))) OR (deep learning)) OR (machine learning)) AND (((((("Macular Degeneration"[Mesh]) OR (macular degeneration)) OR (Macular Dystrophy)) OR (Maculopathy)) OR (Age-Related Macular Degeneration)) OR (Age-Related Maculopathy))) AND ((("Geographic Atrophy"[Mesh]) OR (Geographic Atrophy)) OR (Dry Macular Degeneration))) AND ((((((("Optical Imaging"[Mesh]) OR (Optical Imaging)) OR (colour fundus photography)) OR (Fundus autofluorescence imaging)) OR (Near-infrared reflectance imaging)) OR (Optical coherence tomography)) OR (Optical coherence tomography angiography))

**2. Embase search strategy**

#1 'artificial intelligence':ab,ti OR 'computer reasoning':ab,ti OR ai:ab,ti OR 'machine intelligence':ab,ti OR 'computational intelligence':ab,ti OR 'computer vision system':ab,ti OR 'knowledge acquisition':ab,ti OR 'knowledge representation':ab,ti OR computer:ab,ti OR 'deep learning':ab,ti

#2 'macular degeneration':ab,ti OR 'macular dystrophy':ab,ti OR maculopathy:ab,ti OR 'age-related macular degeneration':ab,ti OR 'age-related maculopathy':ab,ti

#3 'dry macular degeneration':ab,ti OR 'geographic atrophy':ab,ti

#4 'optical imaging':ab,ti OR 'colour fundus photography':ab,ti OR 'fundus autofluorescence imaging':ab,ti OR 'near-infrared reflectance imaging':ab,ti OR 'optical coherence tomography':ab,ti OR 'optical coherence tomography angiography':ab,ti

#5 #1 AND #2 AND #3 AND #4

**3. Web of Science search strategy**

#1 (artificial intelligence) OR (Computer Reasoning) OR (AI) OR (Machine Intelligence) OR (Computational Intelligence) OR (Computer Vision System) OR (Knowledge Acquisition (Computer)) OR (Knowledge Representation (Computer)) OR (deep learning) OR (machine learning )

#2 (macular degeneration) OR (Macular Dystrophy) OR (Maculopathy) OR (Age-Related Macular Degeneration) OR (Age-Related Maculopathy )

#3 (Geographic Atrophy) OR (Dry Macular Degeneration )

#4 (optical imaging) OR (colour fundus photography) OR (fundus autofluorescence imaging) OR (near-infrared reflectance imaging) OR (optical coherence tomography) OR (optical coherence tomography angiography)

#5 #1 AND #2 AND #3 AND #4

**4. Scoups strategy**

((TITLE-ABS-KEY ("optical imaging") OR TITLE-ABS-KEY ("colour fundus photography") OR TITLE-ABS-KEY ("fundus autofluorescence imaging") OR TITLE-ABS-KEY ("near-infrared reflectance imaging") OR TITLE-ABS-KEY ("optical coherence tomography") OR TITLE-ABS-KEY ("optical coherence tomography angiography") )) AND ((TITLE-ABS-KEY ( "geographic atrophy") OR TITLE-ABS-KEY ("dry macular degeneration") )) AND ((TITLE-ABS-KEY ("macular degeneration") OR TITLE-ABS-KEY ("macular dystrophy") OR TITLE-ABS-KEY (maculopathy) OR TITLE-ABS-KEY ("age-related macular degeneration") OR TITLE-ABS-KEY("age-related maculopathy") )) AND (TITLE-ABS-KEY ("artificial intelligence") OR TITLE-ABS-KEY ("computer reasoning") OR TITLE-ABS-KEY (ai) OR TITLE-ABS-KEY ("machine intelligence") OR TITLE-ABS-KEY ("computational intelligence") OR TITLE-ABS-KEY ("computer vision system") OR TITLE-ABS-KEY ("knowledge acquisition" computer) OR TITLE-ABS-KEY ("knowledge representation" computer) OR TITLE-ABS-KEY ("deep learning") OR TITLE-ABS-KEY ("machine learning" ))

**5. Cochrane Library search strategy**

#1 MeSH descriptor: [Artificial Intelligence] explode all trees

#2 ( artificial intelligence) OR (Computer Reasoning) OR (AI) OR (Machine Intelligence) OR (Computational Intelligence) OR (Computer Vision System) OR (Knowledge Acquisition (Computer)) OR (Knowledge Representation (Computer)) OR (deep learning) OR (machine learning )

#3 #1 OR #2

#4 MeSH descriptor: [Macular Degeneration] explode all trees

#5 ( macular degeneration) OR (Macular Dystrophy) OR (Maculopathy) OR (Age-Related Macular Degeneration) OR (Age-Related Maculopathy )

#6 #4 OR #5

#7 MeSH descriptor: [Geographic Atrophy] explode all trees

#8 ( Geographic Atrophy) OR (Dry Macular Degeneration )

#9 #7 OR #8

#10 MeSH descriptor: [Optical Imaging] explode all trees

#11 optical coherence tomography angiography

#12 ("optical imaging"):ti,ab,kw OR (colour fundus photography):ti,ab,kw OR (fundus autofluorescence imaging):ti,ab,kw OR (near-infrared reflectance imaging):ti,ab,kw OR (optical coherence tomography):ti,ab,kw

#13 #10 OR #11 OR #12

#14 #3 AND #6 AND #9 AND #13

**6. CINAHL search strategy**

S1 TX artificial intelligence OR TX Computer Reasoning OR TX Al OR TX Machine Intelligence OR TX Computational Intelligence OR TX Computer Vision System OR TX Knowledge Acquisition(Computer) OR TX Knowledge Representation (Computer) OR TX deep learning OR TX machine learning

S2 TX macular degeneration OR TX Macular Dystrophy OR TX Maculopathy OR TX Age-Related Macular Degeneration OR TX Age-Related Maculopathy

S3 TX Geographic Atrophy OR TX Dry Macular Degeneration

S4 TX Optical Imaging OR TX colour fundus photography OR TX Fundus autofluorescence imaging OR TX Near-infrared reflectance imaging OR TX Optical coherence tomography OR TX Optical coherence tomography angiography

S5 S1 AND S2 AND S3 AND S4
